# Curcumin Prevents Acute Neuroinflammation and Long-Term Memory Impairment Induced by Systemic Lipopolysaccharide in Mice

**DOI:** 10.3389/fphar.2018.00183

**Published:** 2018-03-05

**Authors:** Vincenzo Sorrenti, Gabriella Contarini, Stefania Sut, Stefano Dall’Acqua, Francesca Confortin, Andrea Pagetta, Pietro Giusti, Morena Zusso

**Affiliations:** ^1^Department of Pharmaceutical and Pharmacological Sciences, University of Padova, Padua, Italy; ^2^Department of Agronomy, Food, Natural Resources, Animals and the Environment, University of Padova, Padua, Italy

**Keywords:** lipopolysaccharide, neuroinflammation, curcumin, microglia, sickness behavior, pro-inflammatory cytokines, memory impairment

## Abstract

Systemic lipopolysaccharide (LPS) induces an acute inflammatory response in the central nervous system (CNS) (“neuroinflammation”) characterized by altered functions of microglial cells, the major resident immune cells of the CNS, and an increased inflammatory profile that can result in long-term neuronal cell damage and severe behavioral and cognitive consequences. Curcumin, a natural compound, exerts CNS anti-inflammatory and neuroprotective functions mainly after chronic treatment. However, its effect after acute treatment has not been well investigated. In the present study, we provide evidence that 50 mg/kg of curcumin, orally administered for 2 consecutive days before a single intraperitoneal injection of a high dose of LPS (5 mg/kg) in young adult mice prevents the CNS immune response. Curcumin, able to enter brain tissue in biologically relevant concentrations, reduced acute and transient microglia activation, pro-inflammatory mediator production, and the behavioral symptoms of sickness. In addition, short-term treatment with curcumin, administered at the time of LPS challenge, anticipated the recovery from memory impairments observed 1 month after the inflammatory stimulus, when mice had completely recovered from the acute neuroinflammation. Together, these results suggest that the preventive effect of curcumin in inhibiting the acute effects of neuroinflammation could be of value in reducing the long-term consequences of brain inflammation, including cognitive deficits such as memory dysfunction.

## Introduction

The central nervous system (CNS) immune response (“neuroinflammation”) is emerging as a crucial component in the etiology and course of major neurodegenerative and psychiatric disorders (Glass et al., 2010; Najjar et al., 2013; Amor et al., 2014; Ransohoff, 2016). Microglia, the resident macrophages of the CNS, act as the primary component of an active immune response. Activated upon exposure to endogenous or exogenous injurious stimuli, these cells change their morphology, proliferate, upregulate surface molecules, and become an important source of inflammatory cytokines [e.g., tumor necrosis factor-α (TNF-α), interleukin (IL)-1β, IL-6], chemokines and other mediators [nitric oxide, prostaglandin E2 and cyclooxygenase-2 (COX-2)] that affect CNS functions (Dantzer et al., 2008; Agostinho et al., 2010; Kempuraj et al., 2016). The neuroinflammatory process is normally transient, with activated microglia returning to a resting state once the inflammatory stimulus is resolved. Although an efficient immune response is necessary and critical for host defense, excessive CNS inflammation and over-activation of microglia contribute to neuronal cell damage and exacerbate the severity of neurodegenerative diseases (Norden et al., 2015). In this context, the resolution of an acute inflammatory condition appears crucial to avoid chronic inflammation and ensure appropriate return to tissue homeostasis.

Despite the presence of a blood–brain barrier, bidirectional communication between CNS and peripheral immune system occurs (ThyagaRajan and Priyanka, 2012). Clinical and experimental studies have shown that both chronic and acute systemic inflammatory processes can have deleterious consequences for the brain, representing a risk factor for the development of permanent CNS dysfunction, mostly in the aged and vulnerable brain (Cunningham, 2013; Cunningham and Hennessy, 2015). For example, obesity, diabetes, and atherosclerosis are chronic conditions with inflammatory components that increase the risk of Alzheimer’s disease (Yaffe et al., 2004). Furthermore, acute conditions such as severe sepsis induce microglial activation and brain damage in humans and in rodent models (Cunningham, 2013; Widmann and Heneka, 2014). In recent years, high doses of systemically injected bacterial lipopolysaccharide (LPS; 5–10 mg/kg) have been extensively used in animal models to study the interaction between peripheral inflammation and neurodegenerative disorders. Peripheral LPS induces synthesis of pro-inflammatory mediators in the brain, resulting in a variety of central effects, including synaptic dysfunction, neuronal cell degeneration, and cognitive impairment (Qin et al., 2007, 2013).

Although neuroinflammation is associated with CNS pathologies, clinical trials using anti-inflammatory drugs have been disappointing (Imbimbo et al., 2010). Therefore, growing interest is focused on identifying natural compounds and dietary factors as potential therapeutic agents in neurological disorders associated with inflammation. The natural polyphenol curcumin, the main bioactive component in the rhizome of the turmeric plant (*Curcuma longa*), is a safe and highly pleiotropic molecule with multiple biological targets and a wide range of beneficial activities, thanks to its anti-inflammatory, anti-tumor, anti-oxidative, anti-amyloidogenic, metal-chelating, and cardiovascular protective effects (Hatcher et al., 2008). Many studies have also reported the neuroprotective effect of curcumin in cellular and animal models of Alzheimer’s disease, Parkinson’s disease, Huntington’s disease, multiple sclerosis, depression, and schizophrenia (Lee W.H. et al., 2013). In addition, epidemiological evidence in countries where curcumin consumption is widespread points to a lower incidence of neurodegenerative cases (Ng et al., 2006). The therapeutic benefit of curcumin has been shown mainly after chronic treatment (i.e., weeks or months) (Epstein et al., 2010), whereas short-term administration of curcumin has not been extensively investigated.

In this study, we applied a widely used mouse model of systemic inflammation based on a single intraperitoneal (i.p.) injection of a high dose of LPS to investigate the prophylactic effect of a short curcumin treatment on acute neuroinflammation, as well as its impact on long-term memory impairment.

## Materials and Methods

### Animals

Experiments were carried out using 3-month-old male C57BL/6 mice (Qin et al., 2007), bred in the Animal Facility of the Department of Pharmaceutical and Pharmacological Sciences, University of Padova (originally from Charles River, Lecco, Italy). Mice were housed in individually ventilated plastic cages (3–5 mice per cage) containing pine-chip bedding and paper tubes to provide environmental enrichment, under controlled temperature and humidity, with food and water *ad libitum* on a 12-h light/dark cycle (lights on at 7:00 am). Animal-related procedures were performed in accordance with EU guidelines for the care and use of laboratory animals and those of the Italian Ministry of Health (D.Lg. 26/2014). The study was approved by the Institutional Review Board for Animal Research (Organismo Preposto al Benessere degli Animali, OPBA) of the University of Padova and by the Italian Ministry of Health (Protocol number 722/2015-PR).

### Experimental Procedures

**FIGURE 1:**
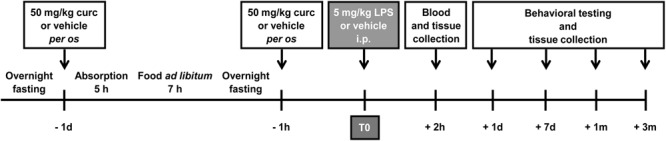
Schematic representation of the experimental design of this study. Mice treated for 2 consecutive days with curcumin (curc; 50 mg/kg) received a single intraperitoneal (i.p.) injection of LPS (5 mg/kg). Control animals received *per os* the same volume of vehicle following the same time schedule and were then injected i.p. with saline. At different times after LPS administration, behavioral tests were performed and blood samples and brain tissues were collected for analysis.

Animals were adapted to handling once daily for at least 5 days before any manipulation. During the handling period, mouse weight and food intake were measured daily. Mice were randomly divided into four experimental groups: control group treated with vehicle (control); curcumin group that received only curcumin treatment (curc); LPS group i.p. injected with LPS (LPS); and LPS pre-treated with curcumin group (LPS + curc). All solutions were prepared fresh on the day of treatment and administered by gavage or i.p. in a final volume of 0.1–0.15 ml. A suspension of curcumin (Sigma-Aldrich, Milan, Italy) in 1% methylcellulose was administered by gavage following periodic fasting once per day at a dose of 50 mg/kg body weight for 2 consecutive days (**Figure 1**). Control animals were treated with the same volume of vehicle according to the same time schedule. On the second day of treatment, LPS and LPS + curc groups received a single i.p. injection of 5 mg/kg of LPS (*E. Coli*, 026:B6; Sigma-Aldrich, Milan, Italy), that was administered 1 h after curcumin or vehicle (**Figure 1**). Animals from the respective control groups received an equal volume of vehicle (sterile, endotoxin-free physiological saline). This dose of LPS is reported to induce an inflammatory response in the brain, behavioral modifications and a progressive neurodegeneration in adult rodents (Qin et al., 2007, 2013; Bossù et al., 2012; Gasparotto et al., 2017). Doses of curcumin up to 100 mg/kg were tested and the lowest effective dose inhibiting the increase in TNF-α mRNA levels induced by systemic LPS was selected for this study (Supplementary Figure 1). Mortality or significant moribundity requiring euthanasia occurred in less than 10% of animals following LPS injection. Animals were assessed in terms of weight, activity in the home cage, and general appearance at 2, 8, and 24 h and daily for 1 week after LPS administration. Behavioral testing and molecular and biochemical analyses were performed at selected time points after LPS challenge (**Figure 1**).

### Behavioral Testing

#### Sickness Behavioral Syndrome

Twenty-four hours after i.p. injection of LPS, the presence of acute sickness behavior symptoms was evaluated by measuring changes in body weight, food intake, and exploratory locomotor activity (Godbout et al., 2005; Davis et al., 2017). The amount of food consumed during the 24-h period was calculated by subtracting the weight of any uneaten pellets (which remained on the cage lid and fell into the cage) at the end of the measured period from that at the beginning.

#### Open Field Test

The open field test was used to assess exploratory locomotor activity. Mice were tested in an apparatus consisting of an opaque open field box (42 cm × 42 cm × 30 cm) constantly illuminated with a white light (25 ± 5 lux). Each mouse was placed alone at the center of the open field arena and allowed to move and explore freely around the arena. During a 10-min session, activity was video-recorded using a camera mounted above the chamber. The open field arena was cleaned with 70% ethanol between each session. Data were analyzed using the ANY-maze^TM^ video tracking system (Stoelting Co., Wood Dale, IL, United States) by a well-trained observer who was blind to the treatment. Behavioral parameters analyzed included: (1) total distance (m) traveled in the arena; (2) time (s) spent in the central zone (the 20 cm × 20 cm inner region) of the testing arena.

#### Novel Object Recognition Test

The novel object recognition test was carried out in the same apparatus as the open field test. The arena and objects were cleaned with 70% ethanol before and at the end of each behavioral evaluation. The task was divided into three different sessions, each lasting 10 min, and carried out for 2 consecutive days. In the first session (habituation session), mice were individually habituated to the open-field apparatus and then returned to their home cage. Twenty-four hours later, during the training session, mice were individually placed in the arena, exposed to two identical plastic objects (rectangular boxes, 3 cm × 3 cm × 6 cm) placed in two corners of the open field apparatus, 8 cm from the sidewalls. The exploration time was video-recorded using a camera mounted above the chamber. Exploration was recorded when the animal touched or reached the objects with the nose at a distance of less than 1 cm. Climbing or sitting on the object was not considered exploration (Frühauf et al., 2015). Immediately after this session, the animal was returned to its home cage. Two hours after the training session, mice were placed back into the arena with two objects: one identical to the familiar object but previously unused to prevent olfactory cues and one novel object of a different shape and color, but similar in size (laboratory flask, 4 cm × 3 cm × 6 cm) (testing session). The time spent exploring each object was recorded. Data were analyzed using the ANY-maze video-tracking system (Stoelting Co.) by a well-trained observer who was blind to the treatment. Animals that failed to complete a minimum of 2 s of exploration during either the training or testing phase were excluded from analysis. The preference index was then calculated, considering the difference in time spent exploring the novel and familiar objects, using the formula:

$$\begin{array}{c} T_{\mathrm{novel}}/\left(T_{\mathrm{novel}}+T_{\mathrm{familiar}}\right) \end{array}$$

The preference index was used as a memory parameter.

### Tissue Collection

At different time points after LPS/saline injection, mice were rapidly euthanized by cervical dislocation, minimizing suffering, discomfort, or stress. To measure curcumin concentration, immediately after sacrifice, blood samples were collected by cardiac puncture for separation of plasma, which was stored at -80°C. Animals were then decapitated, whole brains removed and immediately used or frozen in liquid nitrogen and stored at -80°C until analysis.

To measure mRNA expression of target genes after decapitation, brain regions (cortex, striatum, hippocampus, and cerebellum) were dissected out based on stereological coordinates using a mouse brain atlas (Franklin and Paxinos, 2007; Sorrenti et al., 2018), frozen in liquid nitrogen and stored at -80°C until further analysis.

For immunofluorescence studies, mice were anesthetized with isoflurane and perfused via the cardiovascular system with saline followed by 4% paraformaldehyde (Sigma-Aldrich). Brains were removed intact, post-fixed with 4% paraformaldehyde for 2 h at 4°C, dehydrated through an ascending sucrose series (15 and 30% sucrose) at 4°C overnight and then embedded in Tissue-Tek OCT compound (Sakura Finetek, Torrance, CA, United States). The embedded brains were kept at -80°C until sectioning.

### Liquid Chromatography/Mass Spectrometry (LC/MS)

Plasma and brain tissues were extracted in methanol and analyzed by HPLC/MS using benzanilide as an internal standard. Whole brains were homogenized in 500 μl of methanol and 300 μl of internal standard stock solution (100 μg/ml benzanilide in methanol) and sonicated for 5 min at room temperature. Samples were then centrifuged at 13,000 rpm for 20 min and supernatant was used for analysis. Two-hundred microliter of plasma samples were extracted in 500 μl of methanol and 200 μl of internal standard stock solution. Samples were vortexed and centrifuged at 13,000 rpm for 20 min.

Analysis was carried out using an Agilent 1260 Series HPLC chromatograph equipped with a Prostar 410 autosampler (Varian, Inc., Palo Alto, CA, United States) and coupled with a Varian 320 TQD MS spectrometer (Varian). The mass spectrometer was equipped with an electrospray ionization (ESI) source as the interface and analysis was conducted in positive ion mode. Analyses were performed on a Phenomenex Kinetex EVO C-18 100A, 100 mm × 3mm 5μ column. The mobile phase was (A) water-formic acid (100:2.0 v/v) and (B) acetonitrile. A gradient program was used, as follows: 0 → 1st min A:B (95:5) isocratic; 1 → 15th min: A:B (5:95); 15 → 20th min A:B (5:95) isocratic; 20 → 25th min, return to initial conditions. The mobile phase flow rate was 400 μl/min; the flow was split after the column and 200 μl/min were direct injected in the ESI source. The injection volume was 5 μl.

Quantification was performed using multiple reaction monitoring (MRM) with m/z 369 → 177 transition for curcumin, m/z 449 → 177 for curcumin sulfate, m/z 545 → 177 for curcumin glucuronide and m/z 198 → 105 transition for ISTD benzanilide. The MS parameters were capillary voltage 120 V, needle voltage 4,500 V, shield voltage 350 V, collision energy 15 V, Q1 voltage 1.2 V and Q3 voltage 20.0 V, nebulising gas pressure 15 psi, and drying gas pressure 20 psi.

Curcumin standard solution was prepared at different concentrations 10 μg/ml → 0.1 μg/ml and benzanilide (ISTD) stock solution was diluted 1:10 (50 μg/ml → 0.1 μg/ml). The calibration curve was obtained mixing 500 μl of 0.1 μg/ml ISTD with different volumes (500, 400, 300, 200, 100, and 50 μl) of 0.1 μg/ml curcumin standard solution in order to obtain different curcumin/benzanilide quantity ratios for the calibration curve. Detection limits were 0.09 ng/ml and 0.03 ng/g on plasma and brain tissue, respectively.

### RNA Isolation and Real-Time PCR

Total RNA was extracted from tissues using TRIzol (Invitrogen, Milan, Italy), according to the manufacturer’s instructions. RNA integrity and quantity were determined by RNA 6000 Nano assay in an Agilent Bioanalyzer (Thermo Fisher Scientific, Milan, Italy). Samples were reverse transcribed with Superscript III reverse transcriptase (Invitrogen). The real-time-PCR reaction was performed as described previously (Barbierato et al., 2015). Primer sequences are listed in **Table 1**. The amounts of each gene product were calculated using linear regression analysis from standard curves, demonstrating amplification efficiencies ranging from 90 to 100%. Dissociation curves were generated for each primer pair, showing single-product amplification. Data are presented as specific ratio between the gene of interest and the reference gene (glyceraldehyde-3-phosphate dehydrogenase, GAPDH) normalized to vehicle-treated group (control).

**Table 1:** Primers for real-time PCR used in this study.

Gene target	Primer name	Sequence (5′–3′)
TNF-α	TNF-α For	CAAGTGGAGGAGCAGCTGGA
	TNF-α Rev	CATCGGCTGGCACCACTAGT
IL-1β	IL-1β For	CTGGTGTGTGACGTTCCCATTA
	IL-1β Rev	CCGACAGCACGAGGCTTT
IL-6	IL-6 For	GAGGATACCACTCCCAACAGACC
	IL-6 Rev	AAGTGCATCATCGTTGTTCATACA
COX-2	COX-2 For	GCTGGCCTGGTACTCAGTAGGTT
	COX-2 Rev	CGAGGCCACTGATACCTATTGC
NLRP3	NLRP3 For	CCTGACCCAAACCCACCAGT
	NLRP3 Rev	AGACCTCCCCAATGTGCTCG
CD16	CD16 For	GCAGCAACTTTTCAGCCACA
	CD16 Rev	GAACTGGCGATCCTCCTTCC
GAPDH	GAPDH For	TGGTGAAGGTCGGTGTGAAC
	GAPDH Rev	AATGAAGGGGTCGTTGATGG

### Immunofluorescence and Image Analysis

Using a -20°C cryostat (Leica Microsystems, Wetzlar, Germany), frozen tissues were sliced into 14-μm coronal sections, mounted onto Superfrost glass slides (Thermo Fisher Scientific, Milan, Italy) and stored at -20°C until use. Non-specific staining was blocked by incubating with 5% normal goat serum and 0.1% Triton X-100 in PBS for 1 h at room temperature. Sections were then incubated sequentially with anti-ionized calcium binding adaptor molecule 1 (Iba1) primary antibody (1:800; Wako Pure Chemical Industries, Ltd., Japan) for 2 h, followed by Alexa Fluor 488 fluorescent-conjugated secondary antibody (1:1000; Invitrogen) for 1 h in the above blocking solution (Zusso et al., 2012). Slides were thoroughly washed between steps with PBS. The coverslips were mounted on microscope slides with Fluoromount-G mounting medium (SouthernBiotech, United States). Immunostaining controls included omission of the primary antibody. Images were captured with a confocal laser-scanning microscope (Zeiss LSM 800; Carl Zeiss AG, Germany) and microscope settings were kept constant for all images. The Iba1 fluorescence intensity was measured using the ImageJ software (Fiji, version 1.51n; National Institutes of Health, United States), after subtracting background levels. For comparison among different groups, immunofluorescence intensity was quantified within defined areas of the cerebral cortex (Bhat et al., 2017). Microglial cell density was calculated by counting the number of Iba1-positive cells in the images and expressed as cell number/mm^2^. All areas were measured using the ImageJ software. Six sections per mouse from five different mice per experimental group were analyzed and each section was 50 μm from the previous section. All analyses were performed by an investigator blind to the experimental conditions.

### Statistical Analysis

Statistical analysis was performed using GraphPad Software, version 3.03 (GraphPad Software Inc., La Jolla, CA, United States). Results are expressed as mean ± SEM. Data were analyzed by Student’s *t*-test for comparisons involving two groups or by one-way analysis of variance (ANOVA) followed by Bonferroni’s *post hoc* test for multiple comparisons. A value of *p* < 0.05 was considered to be statistically significant. Additional details are provided in the figure legends, where appropriate.

## Results

### Plasma and Brain Concentrations of Curcumin

Given curcumin’s poor oral bioavailability, we first assessed its ability to reach the CNS for short treatment times. Mice were orally treated with 50 mg/kg curcumin once daily for 2 consecutive days and then i.p. injected with 5 mg/kg LPS or vehicle. Two hours after LPS injection, mice were sacrificed and the plasma and brain tissue analyzed by LC/MS. Brain concentration of curcumin was 41.1 ± 6.7 ng/g. Curcumin metabolism was also examined. Brain levels of the metabolite curcumin sulfate were not significantly different from the concentration of the parent compound. Moreover, low levels of the glucuronide metabolite were detected in brain tissues (3.1 ± 1.6 ng/g). LC/MS analysis revealed that plasma contained very low concentrations of curcumin (8.2 ± 1.8 ng/g), with the sulfate and glucuronide metabolites being more abundant (67.0 ± 10.2 ng/ml and 453.2 ± 110.2 ng/ml, respectively). LPS treatment did not significantly modify brain and plasma levels of either curcumin or its metabolites (**Table 2**).

**Table 2:** Detection of curcumin and its metabolites in brain and plasma.

	Brain			Plasma		
Treatment	Curcumin (ng/g)	Curcumin sulfate (ng/g)	Curcumin glucuronide (ng/g)	Curcumin (ng/ml)	Curcumin sulfate (ng/ml)	Curcumin glucuronide (ng/ml)
Curcumin	41.1 ± 6.7	42.5 ± 5.1	3.1 ± 1.6	8.2 ± 1.8	67.0 ± 10.2	453.2 ± 110.2
Curcumin + LPS	108.3 ± 25.8	35.4 ± 3.0	2.4 ± 0.9	4.8 ± 0.9	35.3 ± 10.8	348.0 ± 32.7

*Data show brain and plasma concentrations of curcumin, curcumin sulfate, and curcumin glucuronide 3 h after administration via gavage. Data are mean ± SEM (n = 3–5 animals/group). Data were analyzed by Student’s t-test [brain curcumin, t_(*5*)_ = 2.17, p = 0.08; brain curcumin sulfate, t_(*8*)_ = 1.72, p = 0.12; brain curcumin glucuronide, t_(*7*)_ = 0.44, p = 0.67; plasma curcumin, t_(*6*)_ = 1.90, p = 0.10; plasma curcumin sulfate, t_(*5*)_ = 2.06, p = 0.09; plasma curcumin glucuronide, t_(*5*)_ = 1.05, p = 0.34].*

### Effect of Curcumin on the Acute CNS Pro-inflammatory Response to Peripheral LPS Administration

**FIGURE 2:**
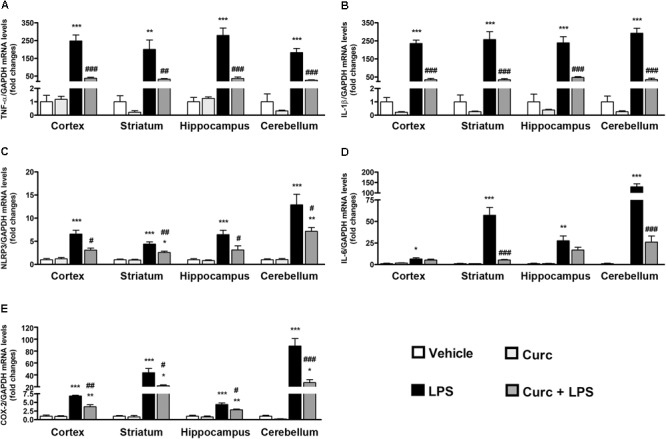
Curcumin inhibits the acute brain pro-inflammatory response to peripheral LPS administration. Mice treated for 2 consecutive days with curcumin (curc; 50 mg/kg) received a single i.p. injection of LPS (5 mg/kg). Two hours following LPS or saline injection, mice were sacrificed and cerebral cortex, striatum, hippocampus, and cerebellum were extracted and prepared as described in Section “Materials and Methods.” **(A)** Analysis of TNF-α mRNA expression levels was conducted via real-time PCR. Results are expressed as fold-increase with respect to control (vehicle only). Data are means ± SEM (*n* = 4 mice/group). Data were analyzed by one-way ANOVA [*F*_(15,48)_ = 32.89, *p* < 0.0001] followed by Bonferroni’s multiple comparison test. ^∗∗∗^*p* < 0.001 vs. control group; ^##^*p* < 0.01 and ^###^*p* < 0.001 vs. LPS-stimulated group. **(B)** Analysis of IL-1β mRNA expression levels was conducted via real-time PCR. Results are expressed as fold-increase with respect to control (vehicle only). Data are means ± SEM (*n* = 4 mice/group). Data were analyzed by one-way ANOVA [*F*_(15,48)_ = 59.32, *p* < 0.0001] followed by Bonferroni’s multiple comparison test. ^∗∗∗^*p* < 0.001 vs. control group; ^###^*p* < 0.001 vs. LPS-stimulated group. **(C)** Analysis of NLRP3 mRNA expression levels was conducted via real-time PCR. Results are expressed as fold-increase with respect to control (vehicle only). Data are means ± SEM (*n* = at least 4 mice/group). Data were analyzed by one-way ANOVA [*F*_(15,54)_ = 26.21, *p* < 0.0001] followed by Bonferroni’s multiple comparison test. ^∗∗∗^*p* < 0.001 vs. control group; ^#^*p* < 0.05 and ^##^*p* < 0.01 vs. LPS-stimulated group. **(D)** Analysis of IL-6 mRNA expression levels was conducted via real-time PCR. Results are expressed as fold-increase with respect to control (vehicle only). Data are means ± SEM (*n* = 4 mice/group). Data were analyzed by one-way ANOVA [*F*_(15,48)_ = 49.62, *p* < 0.0001] followed by Bonferroni’s multiple comparison test. ^∗^*p* < 0.05, ^∗∗^*p* < 0.01 and ^∗∗∗^*p* < 0.001 vs. control group; ^###^*p* < 0.001 vs. LPS-stimulated group. **(E)** Analysis of COX-2 mRNA expression levels was conducted via real-time PCR. Results are expressed as fold-increase with respect to control (vehicle only). Data are means ± SEM (*n* = 4 mice/group). Data were analyzed by one-way ANOVA [*F*_(15,48)_ = 45.03, *p* < 0.0001] followed by Bonferroni’s multiple comparison test. ^∗∗^*p* < 0.01 and ^∗∗∗^*p* < 0.001 vs. control group; ^#^*p* < 0.05 and ^##^*p* < 0.01 and ^###^*p* < 0.001 vs. LPS-stimulated group.

To evaluate the effect of curcumin on the LPS-induced CNS immune response, the mRNA expression of several key pro-inflammatory mediators, such as TNF-α, IL-1β, NLRP3 inflammasome, IL-6, and COX-2 was evaluated in different brain areas, as an index of inflammation. Mice were pre-treated with curcumin once daily for 2 days before a single i.p. injection of LPS. All tested mRNAs increased 2 h following LPS injection in the cortex, striatum, hippocampus, and cerebellum (black bars, **Figure 2**). Pre-treatment with curcumin reduced the LPS-induced increased levels of TNF-α, IL-1β, NLRP3 inflammasome, and COX-2 in all brain areas, while reducing the expression of IL-6 to more selected regions (striatum and cerebellum) (dark gray bars, **Figure 2**), suggesting a protective effect of curcumin against LPS-induced brain inflammation. mRNA expression did not differ between vehicle- (white bars, **Figure 2**) and curcumin-treated (light gray bars, **Figure 2**) mice. Starting from 24 h after LPS administration, mRNA expression levels for all genes in LPS-treated mice returned to control levels (data not shown), suggesting a resolution of inflammation.

### Effect of Curcumin on Acute Microglial Activation in Response to Peripheral LPS Administration

**FIGURE 3:**
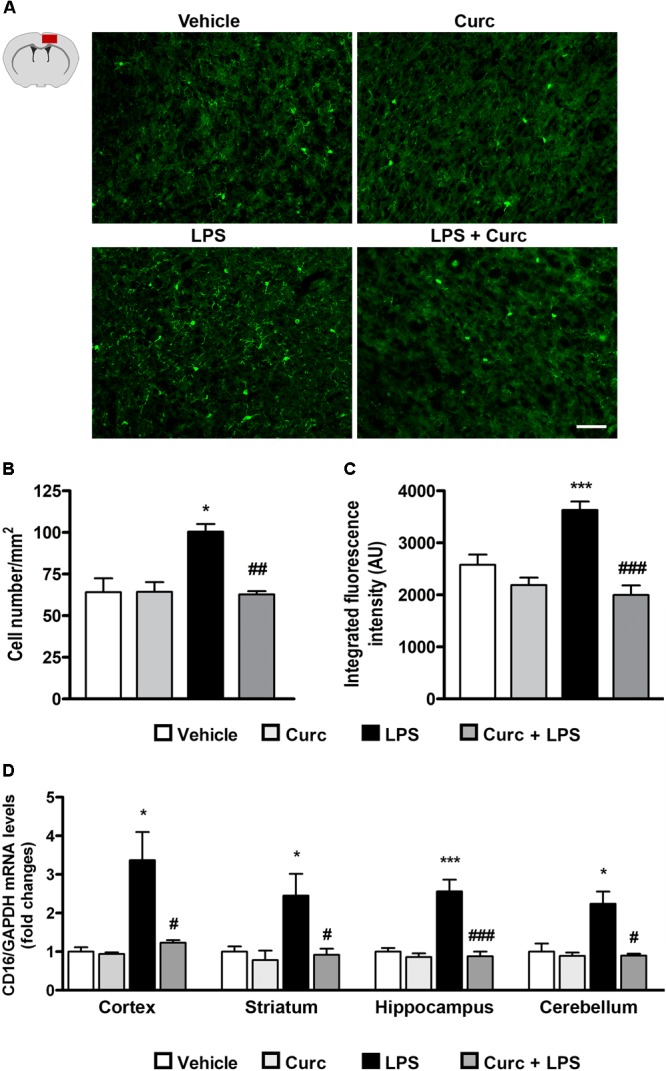
Curcumin inhibits acute microglial cell activation in response to peripheral LPS administration. Mice treated for 2 consecutive days with curcumin (curc; 50 mg/kg) received a single i.p. injection of LPS (5 mg/kg). Two hours following LPS or saline injection, mice were sacrificed and the whole brain, cerebral cortex, striatum, hippocampus, and cerebellum were extracted and prepared as described in Section “Materials and Methods.” **(A)** Schematic of the cerebral cortex in the coronal plane indicating the location (red square) of the area examined and representative images of Iba1-stained microglia through the cortex from the four experimental groups. Scale bar, 50 μm. **(B)** Bar graphs representing the quantification of the number of Iba1+ microglia in the cerebral cortex. Data are means ± SEM (*n* = 5 mice/group). Data were analyzed by one-way ANOVA [*F*_(3,16)_ = 17.81, *p* < 0.0001] followed by Bonferroni’s multiple comparison test. ^∗^*p* < 0.05 vs. control group; ^##^*p* < 0.01 vs. LPS-stimulated group. **(C)** Bar graphs representing the quantification of the mean fluorescence intensity of microglia (Iba1+) in the cerebral cortex. Data are means ± SEM (*n* = 5 mice/group). Data were analyzed by one-way ANOVA [*F*_(3,16)_ = 5.52, *p* < 0.0001] followed by Bonferroni’s multiple comparison test. ^∗∗∗^*p* < 0.001 vs. control group; ^###^*p* < 0.001 vs. LPS-stimulated group. **(D)** Mice treated as described above were sacrificed 24 h following LPS or saline injection. Brain areas were extracted and prepared as described in Section “Materials and Methods” and analysis of CD16 mRNA expression levels was conducted via real-time PCR. Data are means ± SEM (*n* = 4 mice/group). Data were analyzed by one-way ANOVA [*F*_(15,64)_ = 11.18, *p* < 0.0001] followed by Bonferroni’s multiple comparison test. ^∗∗∗^*p* < 0.001 vs. control group; ^#^*p* < 0.001 vs. LPS-stimulated group ^∗^*p* < 0.05 and ^∗∗∗^*p* < 0.001 vs. control group; ^#^*p* < 0.05 and ^###^*p* < 0.001 vs. LPS-stimulated group.

Upon activation, microglia alter their morphology and functional properties in response to environmental changes, a process that is a hallmark of neuroinflammation (Marshall et al., 2013). Since microglial activation is associated with up-regulation of the constitutively expressed marker Iba1, its expression was analyzed in the cerebral cortex 2 h following LPS challenge. The number of Iba1-expressing cells displaying a morphology associated with activated microglia increased after LPS treatment, whereas curcumin pre-treatment completely prevented this effect (**Figures 3A,B**). These results were supported by the quantification of Iba1 staining intensity (**Figure 3C**).

Another feature of pro-inflammatory microglial cell activation is increased expression of the cluster of differentiation 16 (CD16), an M1-phenotype marker (Fumagalli et al., 2015; Yang et al., 2017). As expected, LPS induced an increase in CD16 mRNA expression, which returned to control value with curcumin treatment (**Figure 3D**).

### Effect of Curcumin on Acute Sickness Behavior Syndrome Induced by Peripheral LPS Administration

**FIGURE 4:**
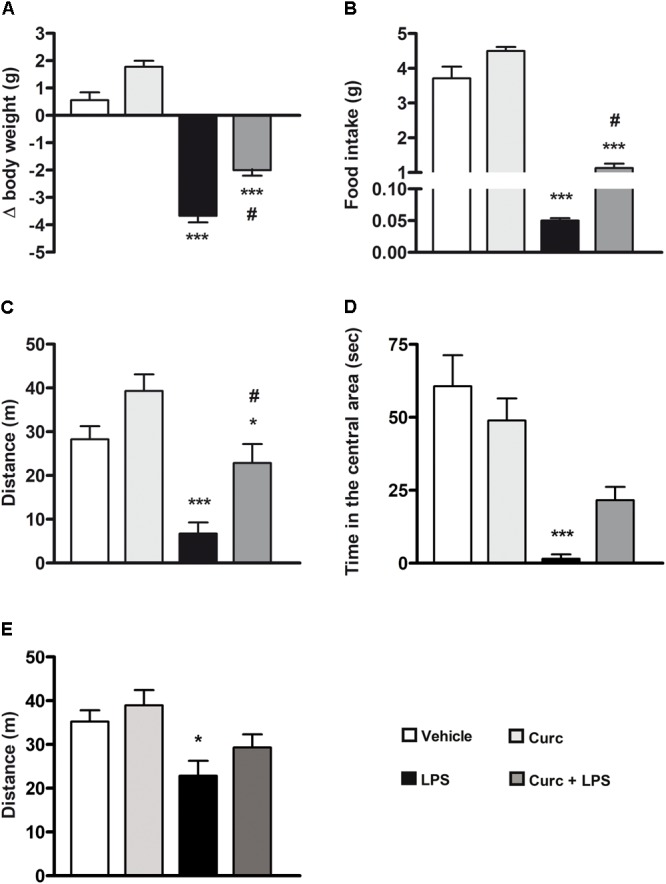
Curcumin attenuates the acute symptoms of sickness behavior caused by peripheral LPS administration. Mice treated for 2 consecutive days with curcumin (curc; 50 mg/kg) received a single i.p. injection of LPS (5 mg/kg). **(A)** Twenty-four hours following LPS or saline injection, body weight was measured as described in Section “Materials and Methods.” Data are means ± SEM (*n* = at least 7 mice/group). Data were analyzed by one-way ANOVA [*F*_(3,28)_ = 97.03, *p* < 0.0001] followed by Bonferroni’s multiple comparison test. ^∗∗∗^*p* < 0.001 vs. control group; ^#^*p* < 0.05 vs. LPS-stimulated group. **(B)** Twenty-four hours following LPS or saline injection, the amount of food intake was measured as described in Section “Materials and Methods.” Data are means ± SEM (*n* = 7 mice/group). Data were analyzed by one-way ANOVA [*F*_(3,24)_ = 136.30, *p* < 0.0001] followed by Bonferroni’s multiple comparison test. ^∗∗∗^*p* < 0.001 vs. control group; ^#^*p* < 0.05 vs. LPS-stimulated group. **(C)** Twenty-four hours following LPS or saline injection, total distance traveled was measured as described in Section “Materials and Methods.” Data are means ± SEM (*n* = at least 7 mice/group). Data were analyzed by one-way ANOVA [*F*_(3,34)_ = 10.98, *p* < 0.0001] followed by Bonferroni’s multiple comparison test. ^∗^*p* < 0.05 and ^∗∗∗^*p* < 0.001 vs. control group; ^#^*p* < 0.05 vs. LPS-stimulated group. **(D)** Twenty-four hours following LPS or saline injection, amount of time spent in the central area of the open field arena was measured as described in Section “Materials and Methods.” Data are means ± SEM (*n* = 7 mice/group). Data were analyzed by one-way ANOVA [*F*_(3,24)_ = 24.58, *p* < 0.0001] followed by Bonferroni’s multiple comparison test. ^∗∗∗^*p* < 0.001 vs. control group. **(E)** Seven days after LPS treatment, total distance traveled was also measured. Data are means ± SEM (*n* = at least 7 mice/group). Data were analyzed by one-way ANOVA [*F*_(4,43)_ = 4.33, *p =* 0.0094] followed by Bonferroni’s multiple comparison test. ^∗^*p* < 0.05 vs. control group.

Pro-inflammatory cytokines released by activated microglia are responsible for the behavioral symptoms of sickness, including reduced body weight, food and water intake, motor and social activities, and altered cognition that usually are observable during the first 24–48 h after LPS treatment (Kelley et al., 2003; Dantzer et al., 2008; Kang et al., 2017; Zager et al., 2018). Systemic LPS induced a reduction of body weight and food intake 24 h after treatment and curcumin protected against LPS-associated weight loss and anorexia (**Figures 4A,B**). Furthermore, and in agreement with previous studies (Dantzer et al., 2008; Davis et al., 2017), LPS diminished the distance traveled and the amount of time that mice spent in the central area of the open-field arena (black bars, **Figures 4C,D**). Curcumin pre-treatment prevented the reduction of locomotion (dark gray bars, **Figure 4C**) and induced a slight increase, although not statistically significant, in the time spent in the central area (dark gray bars, **Figure 4D**). Seven days after LPS administration, behavioral symptoms of sickness returned to control levels (data not shown), with the exception of distance traveled in the arena that was still significantly reduced (**Figure 4E**).

### Effect of Curcumin on Long-Term Memory Impairment Induced by Peripheral LPS Administration

**FIGURE 5:**
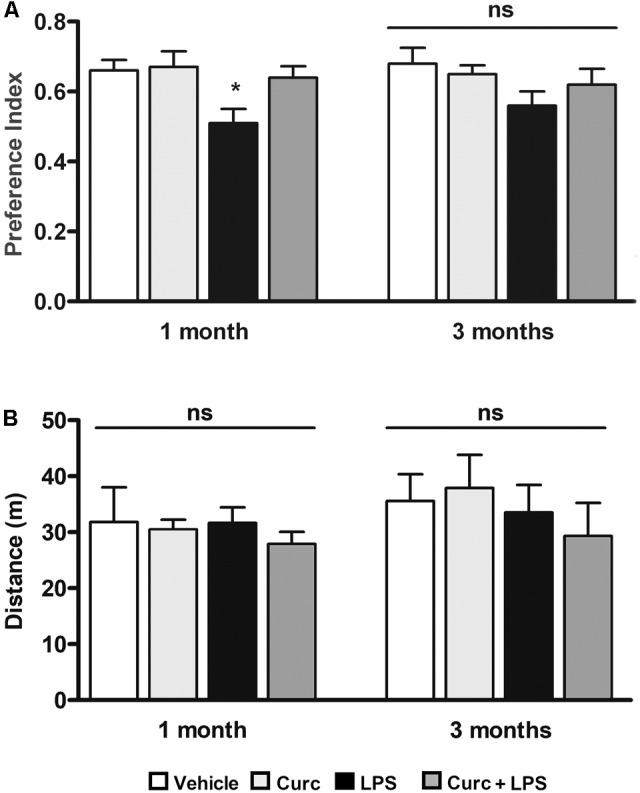
Curcumin inhibits long-term memory deficits induced by peripheral LPS administration. Mice treated for 2 consecutive days with curcumin (curc; 50 mg/kg) received a single i.p. injection of LPS (5 mg/kg). **(A)** One and three months following LPS or saline injection, mice were subject to the novel object recognition test and the preference index, used as a recognition memory parameter, was calculated as described in Section “Materials and Methods.” Data are means ± SEM (*n* = at least 7 mice/group). Data were analyzed by one-way ANOVA [*F*_(7,52)_ = 3.04, *p* = 0.0094] followed by Bonferroni’s multiple comparison test. ^∗^*p* < 0.05 vs. control group. **(B)** One and three months following LPS or saline injection, mice were subject to the open field test to measure total distance traveled in the arena. Data are means ± SEM (*n* = at least 7 mice/group). Data were analyzed by one-way ANOVA [*F*_(7,65)_ = 0.68, *p =* 0.69] followed by Bonferroni’s multiple comparison test. *ns, not significant.*

We next investigated whether curcumin, administered immediately before the LPS treatment, can ameliorate the effect of LPS on long-term memory function, using the novel object recognition test, a widely used model to study memory alterations by measuring recognition memory, attention, and preference for novelty in rodents (Goulart et al., 2010; Antunes and Biala, 2012). One month after treatment, when the acute inflammatory effects of LPS (e.g., mRNA levels of pro-inflammatory markers and sickness behavior symptoms) had completely recovered (data not shown), LPS-treated mice exhibited reduced recognition of a novel object compared to the control mice (left graph, **Figure 5A**). This effect was completely restored 3 months after LPS challenge (right graph, **Figure 5A**). On the other hand, pre-treatment with curcumin improved to control value the recognition memory 1 month after LPS treatment (left graph, **Figure 5A**), suggesting a role of curcumin in preventing the memory impairment induced by peripheral LPS. Since the preference index may be affected by locomotor impairments unrelated to memory function, we monitored the distance traveled in the open field arena that was unmodified by treatments (**Figure 5B**).

## Discussion

Curcumin can interact and modulate the activity of a wide range of target molecules involved in both acute and chronic inflammation (Aggarwal et al., 2007; Aggarwal and Harikumar, 2009). Furthermore, considering that curcumin can cross the blood–brain barrier (Yang et al., 2005; Begum et al., 2008; Vergoni et al., 2009; Tsai et al., 2011; Hoppe et al., 2013), it can modulate the inflammatory response also in the CNS. Using different experimental models of neuroinflammation and neurodegeneration, many studies have evaluated the protective effects of a long-term treatment with curcumin (Liu et al., 2013; Sharma et al., 2017). In contrast, short-term treatments have not been extensively investigated. In the present study, we hypothesized that a short treatment with curcumin, administered to adult mice before an inflammatory challenge, would prevent the effects on neuroinflammation and microglia activation. Since curcumin has poor bioavailability, short serum half-life, and chemical instability in aqueous solution due to the presence of the 1,3-diketone moiety in its structure (Pan et al., 1999; Oliveira et al., 2015; Zusso et al., 2017), we developed an administration protocol to optimize the curcumin pharmacokinetics and possibly the therapeutic effect. Curcumin was administered daily, for 2 consecutive days, via gavage in a 1% methylcellulose suspension, to animals fasted overnight to facilitate oral absorption of curcumin (Begum et al., 2008). A dose of 50 mg/kg of curcumin was selected, based on a dose-response study showing this dose to be the lowest that reduces LPS-induced gene expression of the pro-inflammatory cytokine TNF-α. This dose corresponds to a daily intake of approximately 3–4 g for humans and is considered safe and well-tolerated, as reported by clinical trials showing safety up to 8 g/day of curcumin for 3 months (Chainani-Wu, 2003). LC/MS analysis revealed that curcumin entered brain tissue unmodified 3 h after oral administration, reaching concentrations relevant to its biological activity (Yang et al., 2005). Conversely, at the same time point, the plasma concentration of curcumin was approximately fivefold lower than that of the brain. Vice versa, plasma contained higher concentrations of the sulfate and glucuronide metabolites of orally given curcumin than the brain. In particular, the glucuronide metabolite levels were ∼50-fold higher than those of the parent compound.

The central effect of these brain curcumin levels was evaluated using a standard paradigm of neuroinflammation, based on systemic LPS injection (Kitazawa et al., 2005; Qin et al., 2007). Data on the levels of pro-inflammatory cytokines in the brain, changes in the function and structure of the blood–brain barrier, and neurodegenerative effects after peripheral treatment with LPS are rather discordant in the literature, possibly due to the various experimental protocols used (Catorce and Gevorkian, 2016; Varatharaj and Galea, 2017). Here, we used a single i.p. administration of a high dose of LPS, which stimulated a prompt and transient increase in mRNA expression of pro-inflammatory markers (TNF-α, IL-1β, IL-6, NLRP3, and COX-2) in different brain areas that returned to control levels by 24 h. Mice treated with LPS showed also an acute sickness response that included loss of body weight, anorexia, and reduction of motor activity, as previously reported (Kelley et al., 2003; Dantzer et al., 2008; Kang et al., 2017; Zager et al., 2018). Furthermore, LPS induced anxiety-like behavior. Mice are naturally diffident about entering open or unprotected spaces (Bailey and Crawley, 2009), and the reduced time spent in the central area of the test arena can be interpreted as anxiety-like behavior (Salazar et al., 2012). Further, brain tissues from these mice showed an increased number and reactivity of microglia. Activated microglia have two major phenotypes: M1/pro-inflammatory and M2/anti-inflammatory (Sochocka et al., 2017). M1 activation, involved in the progression of neurodegenerative diseases, causes the release of pro-inflammatory cytokines with increased expression of a cluster of differentiation markers, including CD16 (Yang et al., 2017). Pre-treatment with curcumin prevented LPS-induced M1 microglial cell activation and related sickness behavioral symptoms, except for the anxiety-like behavior. However, further investigation using additional tests of anxiety-like behavior (e.g., elevated plus maze) are needed to better evaluate the effect of curcumin on LPS-induced anxiety.

Curcumin has many molecular targets underlying its anti-inflammatory properties (Aggarwal et al., 2007; Aggarwal and Harikumar, 2009; He et al., 2015), including acting as an inhibitor of the nuclear translocation of the transcription factor NF-κB, which controls the final immune response via expression of several inflammatory genes (Lu et al., 2008; Fu et al., 2014). Furthermore, curcumin could have inhibited the LPS-induced inflammatory response by decreasing expression of Toll-like receptor 4 (Fu et al., 2014), the pattern-recognition receptor that binds LPS and plays a crucial role in the innate immune response (Kawai and Akira, 2010).

Current evidence suggests that inflammatory cytokines lead to memory and cognitive deficits in rodents and humans. LPS can affect memory and cognitive functions in different behavioral paradigms in both rats and mice (Reichenberg et al., 2001; Jacewicz et al., 2009; Ownby, 2010; Bossù et al., 2012; Kawamoto et al., 2013; Lee Y.J. et al., 2013; Zhang et al., 2015). However, these effects have been mostly observed as early consequences of LPS treatment (usually by 7 days), when behavioral symptoms of sickness could be present and locomotor activity reduced, as in our experimental conditions. Here, we observed an impairment of object-recognition memory 1 month after LPS challenge, after complete recovery from sickness behavior and motor dysfunction. In this study, memory deficits were restored by 3 months post-LPS, in contrast with some previous studies which described significant memory deficits 3 and also 10 months post-LPS (Semmler et al., 2007; Bossù et al., 2012). This discrepancy may reflect differences in experimental conditions, such as animal species (rats or mice), age (young vs. aged animals), dose of LPS, site (intravenous or i.p.), and the number of LPS injections or behavioral paradigms used. In particular, Semmler et al. (2007) and Bossù et al. (2012) used rat models of sepsis and described memory deficits up to 3 and 10 months after LPS injection, respectively. To the best of our knowledge, the present findings are the first to show that short-term treatment with curcumin, administered immediately before LPS challenge, anticipates the recovery from delayed memory dysfunction.

Collectively, our results show that a short treatment with curcumin has a preventive effect on both LPS-induced acute neuroinflammation and long-term memory impairment, suggesting that the inhibitory effect on the neuroinflammatory response may reduce consequent cognitive deficits. These results support the potential usefulness of curcumin for treatment of memory and other cognitive deficits evident in many CNS disorders.

## Author Contributions

VS, PG, and MZ designed the experiments. VS, GC, SS, SD, and FC performed the experiments. VS, SD, AP, PG, and MZ analyzed the data. VS and MZ wrote the manuscript. All authors read and approved the final manuscript.

## Conflict of Interest Statement

The authors declare that the research was conducted in the absence of any commercial or financial relationships that could be construed as a potential conflict of interest.
