# Supplementary material for: Curcumin Prevents Acute Neuroinflammation and Long-Term Memory Impairment Induced by Systemic Lipopolysaccharide in Mice
Source: Front Pharmacol. 2018 Mar 5;9:183. doi: 10.3389/fphar.2018.00183 (PMC5845393; doi:10.3389/fphar.2018.00183)
Supplement: Supplementary file 1 [file Data_Sheet_1.DOC]

**SUPPLEMENTARY FIGURE 1**

Fifty mg/kg of curcumin is the lowest dose able to significantly reduce mRNA expression levels of the pro-inflammatory cytokine TNF-α. Mice treated for two consecutive days with increasing dose of curcumin (curc; 10-100 mg/kg) received a single i.p. injection of LPS (5 mg/kg). Two hours following LPS or saline injection, mice were sacrificed and cerebral cortex, striatum, hippocampus and cerebellum were extracted and prepared as described in methods. Analysis of TNF-α mRNA expression levels was conducted via real-time PCR. Results are expressed as fold-increase with respect to control (vehicle only). Data are means ± SEM (n = 4 mice/group). Data were analyzed by one-way ANOVA (F_(31,96)_ = 42.35, *p* < 0.0001) followed by Bonferroni’s multiple comparison test. ***p* < 0.01 and ****p* < 0.001 *vs.* control group; ^##^*p* < 0.01 and ^###^*p* < 0.001 *vs.* LPS-stimulated group.
