# Supplementary figures and images for: Curcumin Prevents Acute Neuroinflammation and Long-Term Memory Impairment Induced by Systemic Lipopolysaccharide in Mice
Source: Front Pharmacol. 2018 Mar 5;9:183. doi: 10.3389/fphar.2018.00183 (PMC5845393; doi:10.3389/fphar.2018.00183)

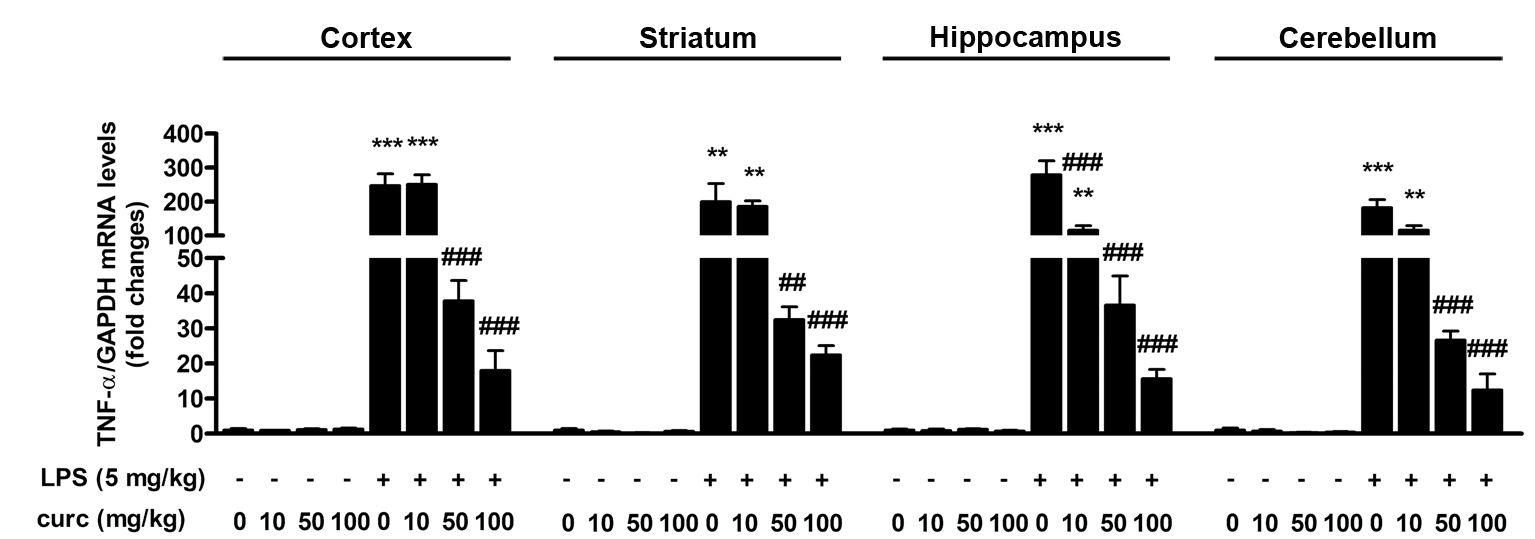

Supplement: Supplementary file 2 [file Image_1.TIF]
